# Editorial: Cytoskeleton Dynamics as Master Regulator of Organelle Reorganization and Intracellular Signaling for Cell-Cell Competition

**DOI:** 10.3389/fcell.2021.782559

**Published:** 2021-10-28

**Authors:** Noa B. Martin-Cofreces, Francisco Sanchez-Madrid, Pedro Roda-Navarro

**Affiliations:** ^1^Department of Immunology, Hospital Universitario de la Princesa, Universidad Autónoma de Madrid, Instituto de Investigación Sanitaria Princesa (IIS-IP), Madrid, Spain; ^2^Centro de Investigación Biomédica en Red de Enfermedades Cardiovasculares (CIBERCV), Madrid, Spain; ^3^Centro Nacional de Investigaciones Cardiovasculares (CNIC), Madrid, Spain; ^4^Department of Immunology, Ophthalmology and ENT, School of Medicine, Universidad Complutense de Madrid, Madrid, Spain; ^5^12 de Octubre Health Research Institute (Imas12), Madrid, Spain

**Keywords:** cytoskeleton, actin, tubulin, cell-cell competition, adhesion, migration, post-translational modifications

The term cell-cell competition, meaning that cell growth and survival is affected by neighboring cells, was used to describe the consequences of this heterogeneous cell environment unveiled through the study of genetic mosaics of *Drosophila melanogaster* (Morata and Ripoll, 1975). In this regard, the organization of multicellular organisms relies on cell–cell interactions involving possible competition between individual somatic cells (Belardi et al., 2020). For example, many neurons compete for the same target to survive during the development of the nervous system (Buss et al., 2006) and the viability of thymocyte clones depends on the establishment of specific cell interactions for the engagement of the correct antigen (Kurd and Robey, 2016). Cell-cell competition may rely on regulators of cell signaling, gene expression or the cytoskeleton, such as vav1 (Tybulewicz et al., 2003), WASp and N-WASp (Cotta-de-Almeida et al., 2007). During the organization of the immunological synapse (IS), a proper regulation of actin and tubulin cytoskeletons is required to achieve full activation, thereby orchestrating the organization of the receptors and organelles essential for effector functions (Martín-Cófreces et al., 2011).

In this collection of articles (Figure 1), Lachowski et al. show that G-Protein-coupled Estrogen Receptor (GPER) activation down-regulates actin dynamics through RhoA phosphorylation at Ser188 and binding to Rho-GDI. The RhoA/mDia pathway is preferentially used by GPER, rather than ROCK/myosin-II, facilitating stress fiber and lamellipodia disorganization in fibroblasts. These data indicate that estrogens can regulate the actin cytoskeleton stiffness, modifying the cell shape and fitness, and point to differential regulation of cell adhesion and migration on different substrates depending on relative cell expression of GPER. Different receptors control actin organization and mechanotransduction in cells, which is now known to affect gene expression through factors such as MRTF/SRF (myocardin-related transcription factor/serum response factor) (Esnault et al., 2014) and YAP/TAZ [Yes-associated protein (YAP) and its homolog transcriptional co-activator with PDZ-binding motif (TAZ, also called WWTR1)] (Dupont et al., 2011). In this regard, Antón and Wandosell review the role of WIP and YAP/TAZ in the connection of the actin cytoskeleton and the development of the nervous system. The role of nuclear *vs*. cytoplasmic actin is discussed in the context of the YAP transcriptional pathway regulation during neurite development and transformation of astrocytes into glioblastoma. Authors conclude that WIP regulates nuclear shuttling of MRTF/SRF and YAP/TAP through actin polymerization, and highlight some unclear aspects of the regulation of the YAP/TAZ pathway in neurons, astrocytes and leukocytes. The connection between the actin cytoskeleton and the nuclear envelope determines cell shape (Gruenbaum et al., 2015) and regulates cell ability to migrate through constrained spaces (Lomakin et al., 2020; Venturini et al., 2020).

**Figure 1:**
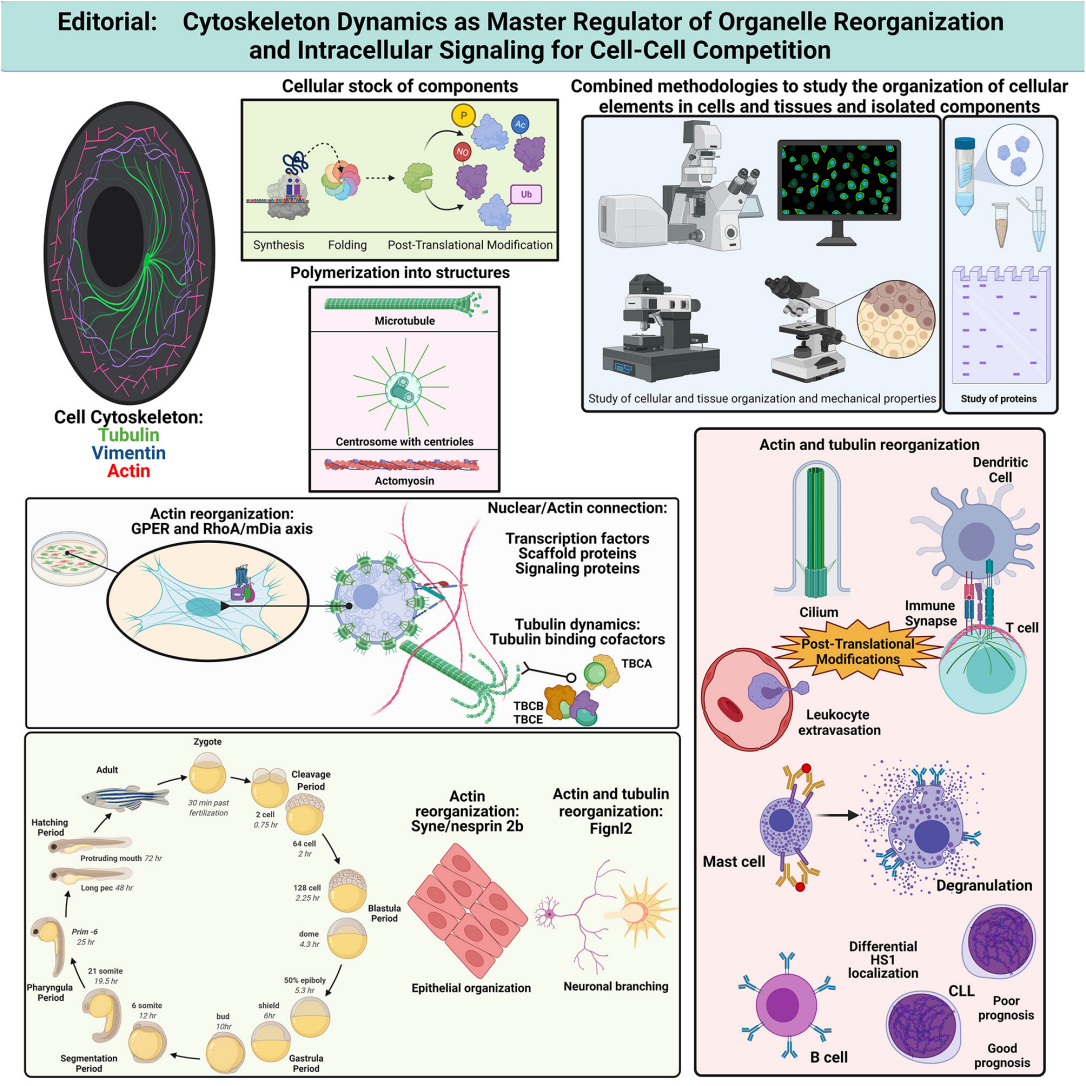
It summarizes the concepts and findings described in the collection of articles pertaining to the Research Topic published in Frontiers in Cell and Developmental Biology, 2021. Created with BioRender.com. Ac, acetylation; CLL, chronic lymphocytic leukemia; Fignl2, fidgetin-like 2; GPER, G-Protein-coupled Estrogen Receptor; HS1, hematopoietic cell-specific lyn substrate-1; mDia, mammalian Diaphanous; NO, S-nitrosylation; P, phosphorylation; RhoA, Ras homolog family member A; TBC, tubulin-binding cofactor; Ub, ubiquitylation.

Li et al. address the study of the protein nesprin-2 (Syne2b), which is an outer nuclear membrane protein that interacts with actin during Zebrafish development (Davidson and Cadot, 2021). Maternal Syne2b/nesprin-2 is required to preserve the epithelial integrity during blastoderm formation. Embryos with defective *Syne2b*/nesprin-2 show delayed progression of the epiboly due to F-actin defective organization. F-actin appears concentrated at multiple cell contacts in defective embryos instead of organizing into the usual polygonal shape. Dong et al. describe a regulatory role for the microtubule severing protein fidgetin-like 2 (Fignl2) in endothelial and neuronal cell branching during Zebrafish development. Sampietro et al. identify by STED microscopy a differential distribution of hematopoietic cortactin homolog HS1 in B cells from chronic lymphocytic leukemia (CLL) patients with poor prognosis. CLL cells show accumulation of HS1 at central regions of the cell in addition to the adhesive basal region observed in cells from healthy or CCL patients with good-prognosis. At adhesion sites, an interaction with vimentin is detected through FLIM-FRET assays. Therefore, the use of super-resolution techniques and sensors of proximity allows finding subtle, though relevant, changes in the molecular organization of the cell that might be the basis of new tumor-specific diagnosis and therapies.

Calvo and Izquierdo discuss the qualitative and quantitative differences in the organization of actin-based structures, such as actomyosin arcs, lamellipodia, or filopodia, at different areas on the T cell side of the IS in the context of secretion. During IS assembly, immune cells reorganize their membranes and organelles. The role of actin cytoskeleton in organizing the IS is still matter of study in different immune cells—T, B, or NK cells arrange their receptors and organelles for directed secretion (Soares et al., 2013; Martín-Cófreces et al., 2014). In this context, Capitani et al. recapitulate the current knowledge on the regulation of endosome function and their regulation by actin dynamics at the IS, as well as their ability to induce actin polymerization. This review article highlights the role of endosomes in the recycling of receptors, as well as in promoting long-term T cell activation.

On the other side of the IS, the actin dynamics also plays relevant roles, which are discussed by Rodríguez-Fernández and Criado-García. This review shows that the organization of the actin cytoskeleton in dendritic cells is relevant to allow correct T cell activation. Immune responses rely on the secretion of mediators, which can be pro-inflammatory. In the case of mast cell degranulation, they trigger allergic responses. In this regard, Ménasché et al. review the coordination of cytoskeletal dynamics and the secretory machinery during stress granule secretion induced by FcεR activation upon allergen engagement. FcεR signals through pathways leading to actin and microtubule reorganization, which resembles the process observed after T and B cell activation.

The regulation of actin dynamics by post-translational modifications (PTMs) is approached by Bago et al.. These authors review the role of nitric oxide and electrophilic cyclopentenone prostaglandins in PTMs of actin and actin binding proteins that facilitate actin depolymerization, ultimately reducing cell adhesion and motility. The effect of these PTMs is discussed in the context of cell-cell communication during endothelial modification to facilitate lymphocyte transmigration and IS formation. The IS and the cilia share a number of features and components (Finetti et al., 2015; Stephen et al., 2018). Primary cilia are indispensable for embryonic development and cell differentiation, which endows ciliopathies with great relevance (Reitter and Leroux, 2017). May et al. address phosphorylation and ubiquitylation of different components during cilia assembly and disassembly. K-63 linked α-tubulin poly-ubiquitylation—which takes place during microtubule de-polymerization (Wang et al., 2019), is used by IFT-A (intraflagellar transport complex A) for retrograde transport during cilia disassembly. Nolasco et al. study the effect of colchicine, a drug used to treat inflammatory diseases such as gouty arthritis and pericarditis. Tubulin binding cofactors (TBCs; TBCA, TBCB, and TBCE) are chaperones involved in the stabilization of the αβ-tubulin heterodimers. Here, authors observe that colchicine prevents the formation of β-tubulin/TBCA complexes by blocking the disassembly of TBCE/TBCB/αβ-tubulin complex. This system is key to regulate the critical concentration of αβ-tubulins needed to promote microtubule assembly. Therefore, colchicine would prevent microtubule dynamics by avoiding recycling of the αβ-tubulin heterodimers, which makes cells more dependent on new synthesis and possible metabolic constraints.

Altogether, this collection of articles summarizes part of the knowledge on cytoskeletal dynamics influencing cell-cell communication involved in sensing changes in the environment supporting development and cell responses. The underlying molecular mechanisms that account for the regulation of cell-cell competition are still barely understood. The diverse regulatory pathways exposed here support a unifying hypothesis postulating that the sensing of extracellular cues through membrane receptors stimulates changes in the cytoskeleton that eventually allow reorganizing other cellular components to adapt to the microenvironment, facilitating an accurate cell response and endurance.

## Author Contributions

NM-C: image composition. NM-C, PR-N, and FS-M: funding acquisition, conceptualization, and writing (original draft, review and editing). All authors contributed to the article and approved the submitted version.

## Funding

This study was supported by grants PDI-2020-120412RB-100 to FS-M and PID2020-115444GB-100 to PR-N from the Spanish Ministry of Economy and Competitiveness (MINECO), grants INFLAMUNE-S2017/BMD-23671 (to FS-M) and Y2018/BIO-5207_SINERGY_CAM (to PR-N) from the Madrid Regional Government, a 2019 grant from the Ramón Areces Foundation Ciencias de la Vida y la Salud and a 2018 grant from Ayudas Fundación BBVA a Equipos de Investigación Científica (to FS-M) and grants PRB3 (IPT17/0019–ISCIII-SGEFI/ERDF), and La Caixa Banking Foundation (HR17-00016 to FS-M). CIBER Cardiovascular (Fondo de Investigación Sanitaria del Instituto de Salud Carlos III and co-funding by Fondo Europeo de Desarrollo Regional FEDER). The Centro Nacional de Investigaciones Cardiovasculares (CNIC) was supported by the Spanish Ministry of Economy and Competitiveness (MINECO) and the Pro-CNIC Foundation. Funding agencies have not intervened in the design of the studies, with no copyright over the study.

## Conflict of Interest

The authors declare that the research was conducted in the absence of any commercial or financial relationships that could be construed as a potential conflict of interest.

## Publisher's Note

All claims expressed in this article are solely those of the authors and do not necessarily represent those of their affiliated organizations, or those of the publisher, the editors and the reviewers. Any product that may be evaluated in this article, or claim that may be made by its manufacturer, is not guaranteed or endorsed by the publisher.
